# Can viral load predict a symptomatic congenital CMV infection? A systematic review and meta-analysis

**DOI:** 10.1007/s00431-025-06015-w

**Published:** 2025-02-11

**Authors:** Serena Salomè, Roberta Gammella, Clara Coppola, Pasquale Dolce, Letizia Capasso, Daniel Blázquez-Gamero, Francesco Raimondi

**Affiliations:** 1 Division of Neonatology, University of Naples Federico II, Via Pansini 5, 80131 Naples, Italy; 2 Department of Translational Medical Sciences, University of Naples Federico II, Via Pansini 5, 80131 Naples, Italy; 3 Pediatric Infectious Diseases Unit, Hospital Universitario 12 de Octubre, Universidad Complutense, Instituto de Investigación Hospital 12 de Octubre (Imas12), Translational Research Network in Pediatric, Madrid, Spain

**Keywords:** Congenital CMV infection, Viral load, Meta-analysis

## Abstract

Cytomegalovirus (CMV) is the most common cause of congenital infection. Although only 10% of infected newborns are symptomatic at birth, a clinical disease may develop later in infancy. An early diagnosis of symptomatic congenital CMV is important for successful treatment. The aim of this study was to evaluate if a higher viral load in different biological fluids at the time of diagnosis correlates with symptomatic disease. A systematic search of Medline, Embase, and SCOPUS from 1976 to August 2024 was performed. Studies were included if the viral load was clearly identifiable as median and mean. Two independent reviewers completed screening, full-text review, data extraction, and quality assessment. Study results were reported as median and interquartile range (IQR: Q1–Q3), with group comparisons based on median differences. Pooled estimates of median differences with 95% confidence intervals were obtained using a median-based meta-analysis approach. Of 4558 studies identified, 11 were used in the meta-analysis with a total of 796 patients (376 symptomatic and 420 asymptomatic babies) for blood determinations and 919 patients for urine (331 symptomatic and 588 asymptomatic babies). Symptomatic infants showed significant higher viral load in blood (pooled difference of median = 1.77 × 10^4^, 0.82;2.72 IU/mL) and a trend in urine (pooled difference of median = 339.7 × 10^4^, − 22.2;701.43 IU/mL).

*Conclusion*: In conclusions, we provide preliminary data that a high CMV load in blood and urine may be associated with symptomatic disease in newborns. Wider and more homogeneous evidence is warranted to confirm our conclusions and to identify a threshold for patients at risk of clinical disease.

**Table:** 

**What is Known**:
• *CMV is the most common cause of congenital infection and an early diagnosis of symptomatic disease is important for successful treatment. A higher viral load in blood was supposed to correlate with symptomatic disease but with non-unique results and data are lacking for different biological fluids.*
**What is New**:
• *We provide preliminary data that a great CMV load in blood and urine may predict newborns at risk of symptoms. Wider and more homogeneous evidence is warranted to confirm our conclusions and to identify a specific viral load threshold for patients at risk of clinical disease.*

## Introduction

Cytomegalovirus (CMV) is a herpes virus responsible of the most common and disabling human congenital infection [1, 2]. A confirmed diagnosis of congenital CMV (cCMV) is based on a positive PCR test performed on a urine sample collected within the first 3 weeks of life. A positive saliva sample should be confirmed with urine because of possible contamination from the maternal genital tract or infected breast milk [3, 4]. Dried blood spots (DBS) routinely collected in the first days after birth can be used for a retrospective diagnosis in children with symptoms referrable to cCMV [5].

Only approximately 10% cCMV infected neonates will present symptoms after birth including bone marrow dysfunction, intrauterine growth restriction, hepatomegaly, variable central nervous system (CNS) involvement, and sensorineural hearing loss (SNHL) [3, 4]. SNHL is detected at birth in 12.7% of infected children and can emerge postnatally in 4.5% [6], can be progressive, and may be partially mitigated through the use of antivirals [7, 8]. Thus, there is a pressing need for precise and early prognostic markers to anticipate sequelae accurately.

Blood viral load has been reported in the literature to be greater in children with symptoms at birth, and children with undetectable or low blood viral load (< 1000 UI/ml) seem to have a better long-term prognosis [9–13]. However, these studies are mainly based on small case series, and some used a heterogeneous definition of symptomatic disease at birth [6–9]. Moreover, a greater viral load in symptomatic children was not confirmed by other studies [14, 15]. The role of individual viral load on other body fluids is even less defined. In the present study, we systematically reviewed the existing literature and performed a meta-analysis on cCMV viral load in different body fluids as a diagnostic tool for symptomatic cCMV.

## Materials and methods

The investigation was performed according to the Preferred Reporting Items for Systematic Reviews and Meta-Analysis (PRISMA) guidelines [16]. Before starting the project, we developed a systematic review protocol, including the choice of databases to be searched, search terms, eligibility criteria, and data to be extracted. Methods to aggregate data and to solve any dispute were also decided. We registered the protocol in the International Prospective Register of Systematic Reviews (ID No. CRD42024537242). The protocol can be accessed by the following link: https://www.crd.york.ac.uk/prospero/display_record.php?RecordID=537242. In addition, it has been also registered on protocols.io as follows: dx.doi.org/10.17504/protocols.io.14egn61qml5d/v1.

Institutional Review Board approval is not required for this type of studies.

Studies were selected according to the following criteria.

### Eligibility criteria

We retrieved all English-language research articles reporting evaluation of viral load in biological samples at time of diagnosis in infants with congenital CMV infection. Moreover, patients should be defined according the severity at birth with classification criteria explicitly reported. Considering that this definition was not overlapping through different articles in past years, we accepted the recent definition of the European Consensus, where isolated SNHL is included in the symptomatic onset, as summarized in Table 1 [17].

**Table 1:** **Symptoms and signs of infants with cCMV** [17]

Clinical symptoms/signs on physical examination
Intrauterine growth restriction (IUGR; birth weight < −2 SD for GA)*
Microcephaly (head circumference (HC) < −2 SD for GA)*†
Petechiae or purpura
Blueberry muffin rash (intradermal hematopoiesis)
Jaundice
Hepatomegaly
Splenomegaly
Abnormal neurological examination (lethargy, hypotonia, seizures, poor suck)
Abnormal laboratory results
Anemia (according to reference hemoglobin and hematocrit values for age and sex)
Thrombocytopenia (< 100,000 per µL)
Leukopenia, isolated neutropenia (< 1,000 per µL)
Elevated liver enzymes (ALT/AST at least 2 times ULN)
Conjugated hyperbilirubinemia (direct bilirubin > 2 mg/dL)
Cerebrospinal fluid (CSF)
Abnormal indices, positive CMV DNA
Neuroimaging
The abnormalities can be classified into two types: 1) Inflammatory or destructive changes resulting from the direct effect of the virus or the immune/inflammatory response: lenticulostriate vasculopathy, germinolytic pseudocysts (caudothalamic, temporal, frontal), occipital horn septations, ventriculomegaly, periventricular calcifications, white matter abnormalities (i.e., increased signal intensity on T2-weighted MRI) 2) Brain developmental disruptions: cortical malformations (typically polymicrogyria or poorly developed sulcation), cerebellar hypoplasia
Hearing evaluation
SNHL (hearing threshold > 20 dB, uni- or bilateral)
Ophthalmologic evaluation
Chorioretinitis or scarring

*ULN* upper limit of normal

*Use ethnic-specific or multi-ethnic growth charts. cCMV is more likely in symmetric intrauterine growth restriction (IUGR), where weight and HC are proportionally affected. This is in contrast to asymmetric IUGR, where HC is preserved while weight is compromised due to reduced fetal nutrition in the late second or third trimesters

†Microcephaly in cCMV can be of two types. In *proportional microcephaly*, due to symmetric IUGR, both HC and weight are < − 2SD and in proportion to each other. By contrast, *relative microcephaly* (i.e., HC *z* score–weight *z* score < − 2) has a high specificity for central nervous system (CNS) involvement and poor neurological outcomes

We selected studies reporting data about viral load in biological samples at time of diagnosis. CMV viral loads can be expressed both in copies per milliliter (cp/mL) or genome equivalents per milliliter (ge/mL) and international units per milliliter (IU/mL) with a conversion factor of 1 [18]. For this reason, studies expressing viral load in both measurements were included. Articles describing viral load on biological samples of children treated with antivirals at the time of sample collection were excluded because the therapy reduces viral load [7].

Viral load in children with symptomatic infection at onset was compared with those with asymptomatic infection. It could be described as median or mean in symptomatic and asymptomatic groups.

Our outcome was to test the accuracy of viral load in different biological samples to differentiate symptomatic and asymptomatic infection in children with cCMV.

The databases PubMed, SCOPUS, BioMed Central, Web of Science, and the Cochrane library were searched until August 2024. We decided not to select a starting time for searching because it was difficult to establish a specific moment when CMV DNA was evaluated in cCMV.

The above databases were searched using keywords: congenital (AND) cytomegalovirus infection, congenital CMV infection. The addition of “viral load” to keywords reduced evaluable studies because not all expressed in the title these measurements. Reference lists of included articles were scanned for any additional eligible studies.

We excluded unpublished or non-peer-reviewed reports. Moreover, we excluded review papers, case reports due to likely reporting bias, and papers in which only symptomatic or asymptomatic infants were described, due to a not possible compare of the two groups in the same population enrolled.

Two reviewers (SS and RG) independently selected eligible abstracts and verified the acceptability of the full studies. Three authors (SS, RG, and PD) extracted data and assessed risk of publication bias (as reported in details in the statistical section). Any disagreements regarding grading of quality were resolved through discussion with a third author (FR). Results were compared and discussed among all the authors and controversies were resolved by discussion.

Data were extracted using a standardized form derived from the Cochrane data collection template and reported in a Microsoft Excel (Microsoft, 2013) spreadsheet. The following data were extracted from the study; author, year of publication, number of children included and number of symptomatic and asymptomatic infants at birth, type of biological sample used for viral load evaluation, viral load in symptomatic and asymptomatic group expressed as median or media value, minimum, max and first and third quartile, and *p*-value.

The methodological quality of the eligible studies was independently assessed by two reviewers using the Quality Assessment of Diagnostic Accuracy Studies 2 (QUADAS-2) tool [19]. The risk of bias was classified as low, high, or unclear. Any discrepancies between the investigators in the quality assessment will be documented and discussed until achieving a consensus. All eligible studies were considered for the meta-analysis, regardless of their quality, and assessed for risk of bias.

### Statistical analysis

Quantitative variables were expressed as median and interquartile range (IQR: Q1-Q3). The viral load was expressed as CMV PCR (copies/mL or IU/mL). For a limited number of studies [20, 21], where results were presented as mean and standard deviation, medians and IQR were calculated assuming the normality of the variable. Median differences will be used for comparisons between groups.

Pooled estimates of median differences were calculated through a median-based meta-analysis approach, using the quantile matching estimation technique as proposed by McGrath et al. [22]. Median differences and their 95% confidence intervals will be presented using forest plots. Heterogeneity among studies was evaluated using Cochran's *Q* test and by Higgins and Thompson *I*^2^ statistic. Heterogeneity was categorized as low for *I*^2^ values below 25%, moderate for values between 25 and 50%, and high for values above 50%.

Publication bias was assessed by inspecting funnel plots for visual asymmetry, positioning comparison-specific effect sizes against their standard errors. Additionally, Egger’s regression test was applied to assess the asymmetry of funnel plots, aiming to determine the statistical significance of the relationship between effect sizes and their corresponding standard errors.

All statistical analyses were conducted using R statistical software. The “metamedian” R package [23] was used for meta-analysis.

Authors assessed each study sampling strategy, representativeness, comparability of the samples, and use of comparable methods of analysis. If applicable, authors could be contacted to clarify unclear or missing observations, data, or outcomes.

The strength of the body of evidence was assessed as follows. For the interpretation of the study contribution to the findings, reference was made to the prior assessment of the included studies’ methodological quality. Moreover, consistency and inconsistency across findings were assessed and any incoherent or contradictory evidence was highlighted and discussed. In addition, GRADE or CERQual approaches were employed for standardized assessment of cumulative evidence quality (as shown in Tables 2 and 3).

**Table 2:**
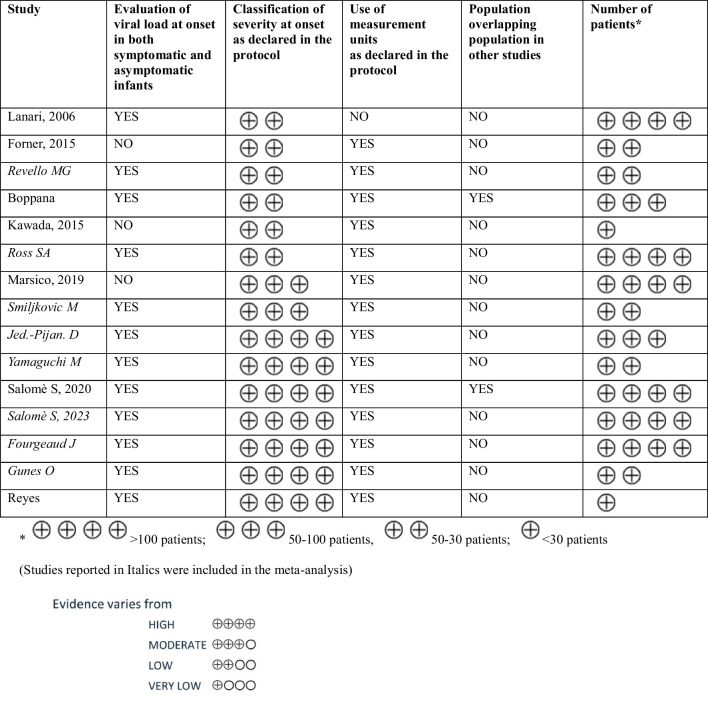
Quality of the evidence (GRADE) for viral load in blood

**Table 3:**
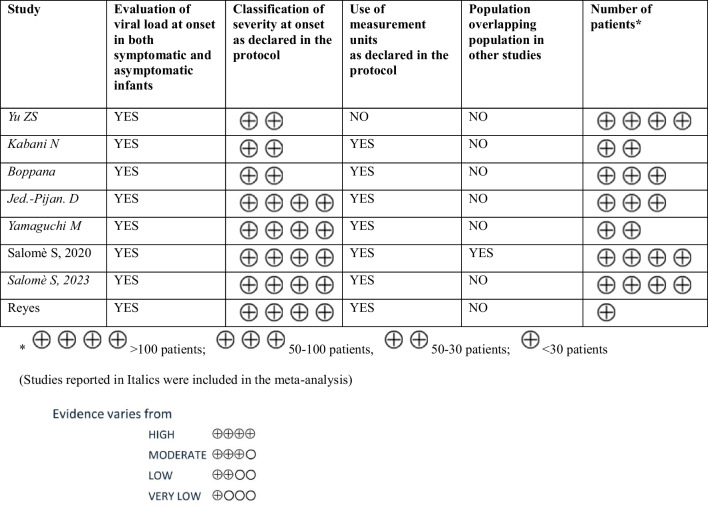
Quality of the evidence (GRADE) for viral load in urine

## Results

**Fig. 1:**
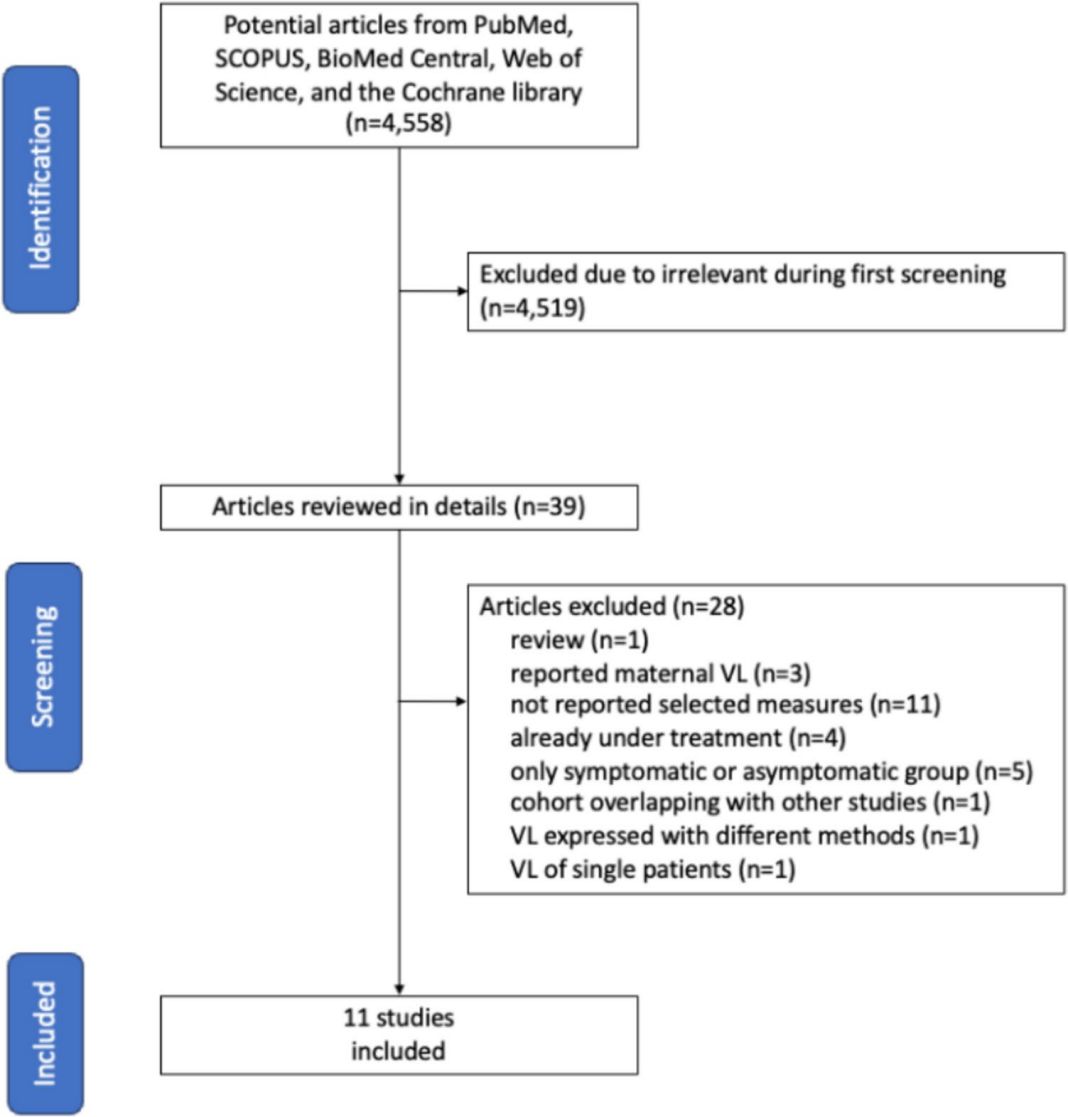
Study selection process

The systematic search identified 4558 articles, of which 4519 were excluded as irrelevant. Full texts of the remaining 39 studies were retrieved for evaluation, and a total of 11 studies met the inclusion criteria. No additional study was identified through a review of the reference lists of these studies. The results of search and study selection process are detailed in Fig. 1.

Considering viral load in *blood*, eight studies were included in the analysis [12, 13, 15, 21, 24–27]. Three studies were excluded because only included data on asymptomatic [11] or symptomatic infants [10, 28]. Other two studies reporting data on both groups and with median value evaluable for analysis were excluded because the described cohort was also included in a following paper [14, 20]. One study demonstrated on a small number of babies that neonatal viremia was an independent predictive factor for clinical disease, but it could not be included because viral load was expressed with units of measure impossible to convert [9].

**Fig. 2:**
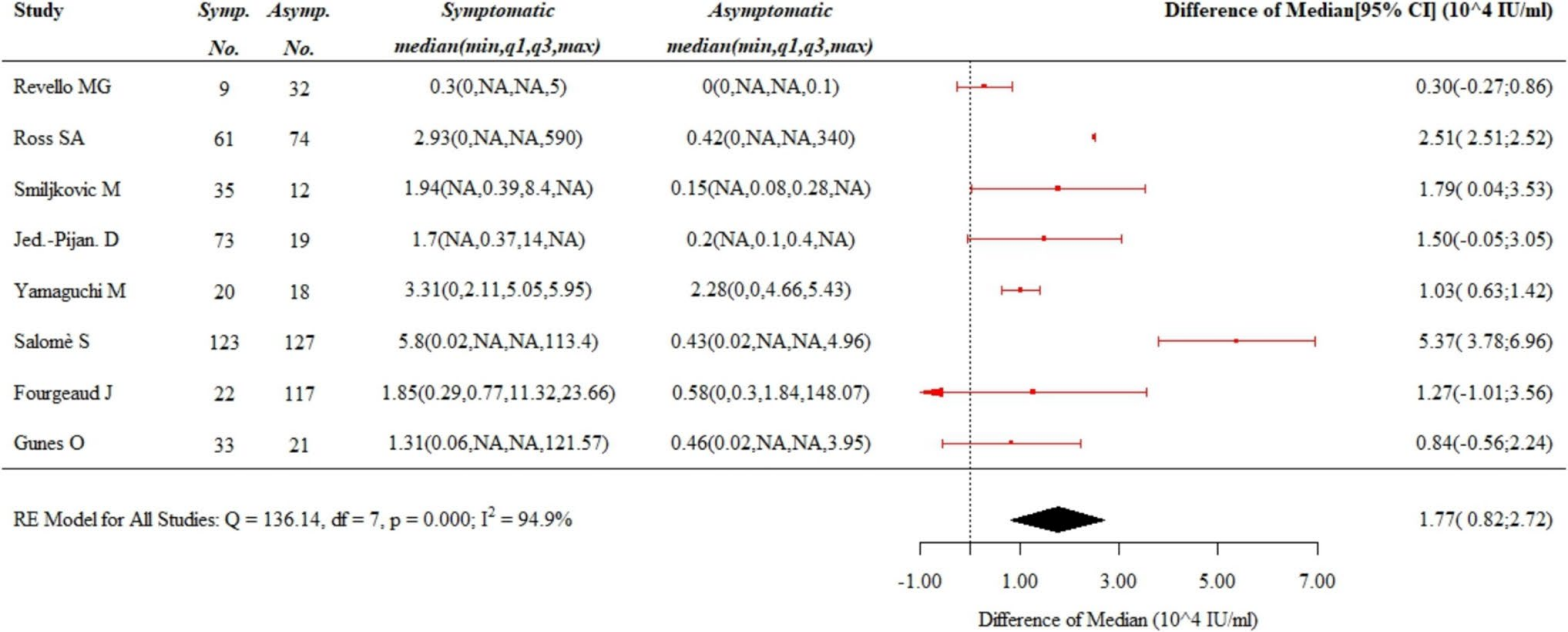
Viral load in blood

Figure 2 reports the individual and collective evidence from the eight studies included in the systematic review.

A total of 796 patients were involved, with 376 of them classified as symptomatic at onset (47% of the population). This group showed significant higher blood viral load with a pooled difference of median of 1.77 × 10^4^. Though data show remarkable heterogeneity, blood viral load is significantly associated with symptomatic disease at onset.

**Fig. 3:**
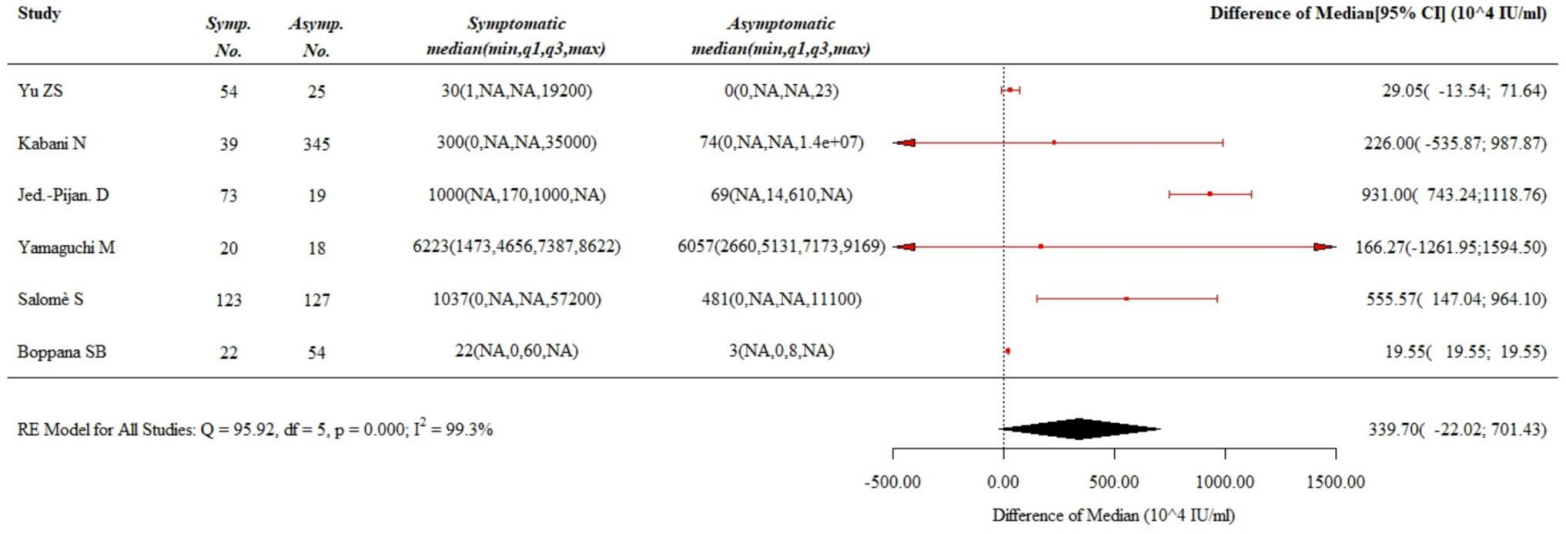
Viral load in urine

Six studies were included in the analysis [15, 20, 21, 27, 29, 30] of CMV load in urine whose results are summarized in Fig. 3.

A total of 919 patients were involved as a whole, with 331 of them classified as symptomatic at onset (36% of the population). The difference between babies with symptoms and those asymptomatic borders the statistical significance with a trend toward a greater urinary viral load (pooled difference of median = 339.7 × 10^4^). Again, the wide dispersion of results in the individual studies precludes a more assertive conclusion.

Regarding CMV viral load in saliva and DBS, we retrieved insufficient data to elaborate even preliminary conclusions.

While *Saliva* is a minimally invasive source of the virus, only three studies were identified reporting viral load [12, 30, 31]. The paper by Xu et al. [31] was excluded as it enrolled only asymptomatic patients. In the two remaining studies, both enrolling a large prevalence of asymptomatic infants came to opposite conclusions. Kabani et al. [30] found a higher viral load in symptomatic babies while Fourgeaud et al. [12] did not.

Three studies [31–33] were identified reporting viral load in *DBS* but the one by Xu et al. [31] was excluded because only asymptomatic newborns were enrolled. The two remaining studies both showed a greater viral load in symptomatic infants although data are insufficient for a meta-analysis.

Another study by Reyes et al. described a small cohort of 16 patients, both symptomatic and asymptomatic at onset, evaluated for viral load in different biologic fluids (blood, urine, saliva, DBS, and cord blood) [34]. Unfortunately, it could not be included in the analysis because data for each specimen in single patients was insufficient for statistical evaluation.

## Discussion

Data on viral load in blood showed that a higher number of CMV copies were associated with symptomatic disease at birth. The analysis on urinary viral load demonstrated an association that bordered statistical significance without reaching it. Usually, a urine sample is the first one collected by a newborn with suspicion of cCMV and its viral load can be as red flag for symptomatic onset.

We speculate that both technical and clinical reasons might account for these results that currently prevent the use of viral load as a useful prognostic guide.

Different laboratory protocols may lead to considerable differences in results [35]. Furthermore, the definition of symptomatic infection has changed over the years. Both Ross et al. [25] and Smiljkovic et al. [26] grouped patients with isolated SNHL with the totally asymptomatic. On the other hand, in the study by Boppana et al. [20], infants now considered as symptomatic were reported in three separate groups (namely “Asymptomatic with hearing loss,” “Symptomatic with hearing loss,” and “Symptomatic with normal hearing”).

Finally, the variable ratio between symptomatic and asymptomatic patients in individual studies might have generated another source of heterogeneity in the results [12, 20, 24, 26, 27, 29, 30].

Insufficient data were found regarding viral load in saliva and DBS to elaborate even preliminary conclusions, both considering number of studies eligible, and number of patients enrolled. Regarding saliva, currently only few studies are available but they should increase in the near future in view of possible universal neonatal screening using this type of biological fluid that is considered more suitable for this purpose based on how simple is to collect this sample. On the other hand, DBS is usually used to diagnose retrospectively a congenital infection in infants with suspected features of cCMV beyond the neonatal period even if in some countries, DBS has been employed for universal screening of congenital cytomegalovirus infection, as it is already used in metabolic screening. Therefore, the viral load in both these samples could play a role as prognostic factor in the future and not only in the diagnosis of cCMV.

Furthermore, some studies proposed a threshold for viral load in blood that could predict the probability of symptomatic disease [20, 25, 26], although different values were identified in different cohort. In fact, in the cohort by Boppana et al., a lower risk for hearing loss was demonstrated in infants with < 10,000 copies/mL [20] while data by Ross et al. [25] about children with asymptomatic infection identified a threshold of < 3500 ge/mL with a negative predictive value of 94.4% and a positive predictive value of 8% and Smiljkovic et al. [26] identified that 100,000 copies/mL were related to a 100% probability of moderate to severely symptomatic disease. We aimed to define a threshold derived by all data included in the meta-analysis, but it was not feasible because the single value of viral load for each patient enrolled in the studies included was not available.

Our meta-analysis has a main limitation that is the heterogeneity of the populations in the different studies included. In fact, a meta-regression or subgroup analysis was not feasible because the different papers described different basic populations features, such as gestational age or other possible confounders. Moreover, the available studies have different sampling and laboratory protocols that surely lead to major differences in results. We tried to reduce these differences selecting studies with more similar laboratory protocols without losing in numerosity of the population analyzed.

In conclusion, the association of a high CMV load in body fluids with symptomatic disease is an appealing tool to orientate clinical decisions. Therefore, current data are promising but not conclusive. Further and more homogeneous evidence might lead to identifying specific prognostic thresholds that can lead to a clinical decision. International registries collecting data about a wider and more homogenous cohort, such as the CCMVNET that is based on standardized laboratory protocols and symptomatic definitions, could fit for these purposes both increasing statistical power and defining a specific viral load threshold for clinical decision. Moreover, using this kind of cohort gives the opportunity to include data on long-term outcomes (e.g., hearing loss, neurological development) to assess the predictive value of viral load more comprehensively, on onset and on sequelae too. Finally, further studies could evaluate if variability in CMV seroprevalence and population genetics influences viral load findings.

## Data Availability

No datasets were generated or analysed during the current study.

## Abbreviations

CMV: Cytomegalovirus
cCMV: Congenital CMV
DBS: Dried blood spots
CNS: Central nervous system
SNHL: Sensorineural hearing loss
PRISMA-P: Preferred Reporting Items for Systematic Reviews and Meta-Analysis Protocols
cp/mL: Copies per milliliter
ge/mL: Genome equivalents per milliliter
IU/mL: International units per milliliter
QUADAS-2: Quality Assessment of Diagnostic Accuracy Studies 2
IQR: Interquartile range
